# Synthetic GNSS spoofing data generation using field recorded signals

**DOI:** 10.1016/j.mex.2018.10.004

**Published:** 2018-10-09

**Authors:** Abdul Malik Khan, Naveed Iqbal, Muhammad Faisal Khan

**Affiliations:** National University of Sciences and Technology, Islamabad, Pakistan

**Keywords:** *GNSS Spoofing Data Synthesis*, Global Navigation Satellite System, GNSS spoofing, Synthetic data generation

## Abstract

With the increase in GNSS user base, the studies of threats and vulnerabilities of GNSS system are also increased. Among the threats, spoofing is of particular interest because of the risk associated with it. The studies on spoofing are generally limited to simulated scenarios as the real world spoofing attack is very difficult to create or spot and record for analysis. This paper presents a method of generating baseband spoofing data using real world signals by simultaneous recordings of GNSS signals using two separate receivers, where one of them simulates the receiver under attack and the other simulates the response the spoofer will be going to produce to fabricate the attack. After taking the records and merging them to create the spoofing baseband signals, it is checked against several spoofing detection methods to verify the valid spoofing attack being present in the signal. This method produces the signal recordings that have real world disturbances in it that may be difficult to simulate.

The developed method has the following advantages:

• It does not require very expensive hardware to produce an intermediate spoofing signal.
• The user has control over the spoofing power advantage.
• The same scenario can be reproduced with varying parameters.

## Graphical abstract

**Figure:**
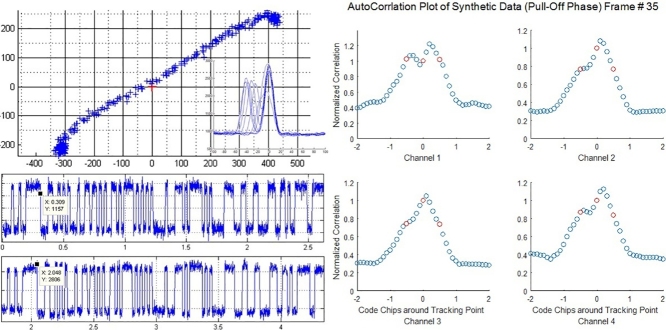

Specifications Table

**Table:** 

Subject area	• *Engineering*
More specific subject area	*Global Navigation Satellite System*
Method name	*GNSS Spoofing Data Synthesis*
Name and reference of original method	*Simulated GNSS Spoofing:* T. E. Humphreys, B. M. Ledvina, M. L. Psiaki, B. W. O’Hanlon, and P. M. Kintner, Jr., “Assessing the spoofing threat: Development of a portable GPS civilian spoofer,” in Proc. ION GNSS, Savannah, GA, 2008, pp. 2314–2325.
Resource availability	*Software GNSS Receiver*

## Method details

### Synthetic generation of spoofing signal

Global Navigation Satellite Systems (GNSS) are becoming primary source of Position, Navigation and Timing (PNT) for a variety of applications and has a big user base [2] [3]. The GNSS signals are vulnerable to environmental effects, interference, jamming, and spoofing due to their low power and open signal structure [1] [5] [6]. With a greater user base and open architecture, it is becoming a tempting target for the attackers to spoof the GNSS signal. To overcome the vulnerability of receiver, anti-spoofing techniques are also under studies. Development of anti-spoofing techniques requires real or simulated signal to verify the effectiveness of the techniques. The development of spoofer or the dataset for the purpose of anti-spoofing is discussed in [4] [7].

**Fig. 1:**
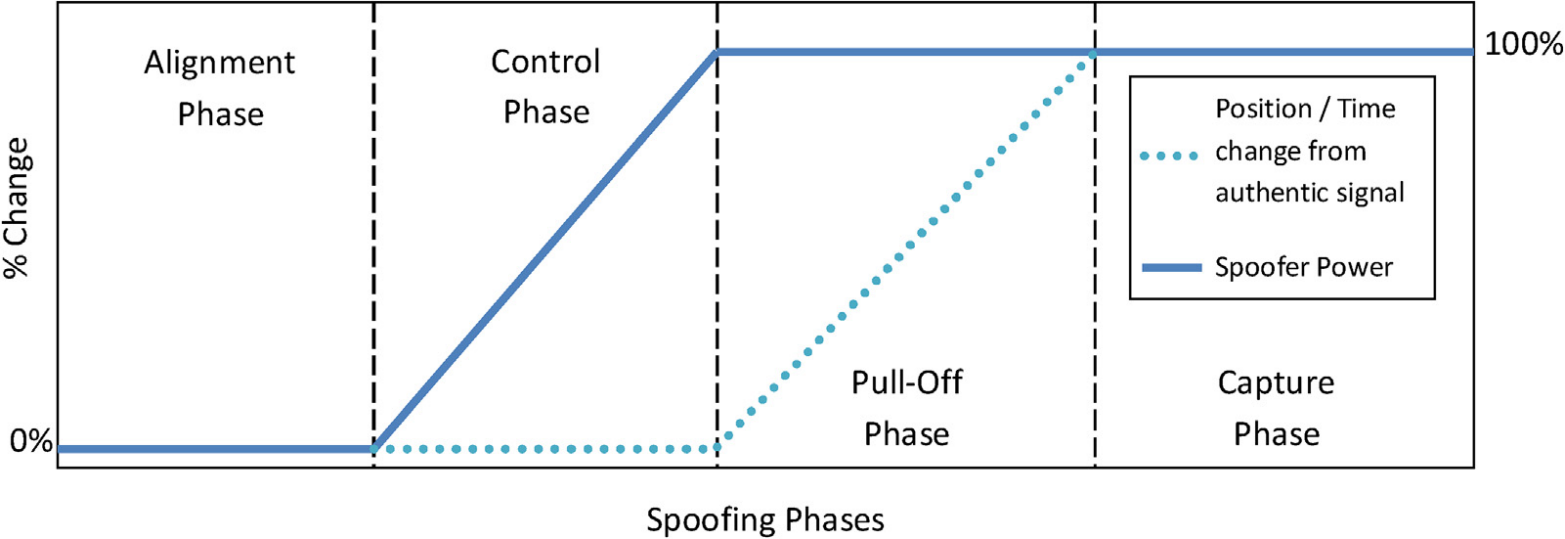
Spoofer delay profile (position/time change) and power profile for each spoofing phase.

In this paper we present a method to synthetically generate the GNSS spoofing signal using the real world field data recorded by two GNSS receivers (spoofer and target). This data also captures the environmental effects because of recording in the actual environment, thus generating more authentic and close to real world spoofing signal. The synthetic spoofing signal was generated by combining two recorded signals and corrected for the code / carrier phase, clock and power matching and spoofer power profile as given in Fig. 1. The spoofing can be classified as time-push or position push based on the spoofer intended modification in target receiver measurement [7].

#### Time-push scenario generation

Time-push scenario is synthesized using a single receiver data for spoofer and target receiver, because it is intended to affect the receiver clock correction term only. To generate time-push scenario, data from single receiver can be delayed and mixed with it, so it does not require code / carrier phase and clock matching. As this is a relatively easier method so it is not discussed in details here.

#### Position-push scenario generation

To synthesize a position-push spoofing scenario, two receivers are employed in field to record the data and corrected and mixed according to the procedure given in the next sections. The spoofer receiver captures the position push information during its movement relative to the target receiver.

#### Position / time control (spoofer delay profile)

The spoofer delay profile should be planned first that formulate the time and change during each phase of the spoofing. Fig. 1 shows one of the planned spoofer delay profile required to generate planned position-push or time-push scenario. The profile contains the change with repost to the target receiver in spoofer power and position (or time) planned in each phase of the spoofing sequence to mimic the intermediate spoofing attack.

A spoofer following the profile given in Fig. 1 does not generate any power during the alignment phase in which it estimates the user state. In control phase, it increases transmit power gradually to the planned level to get the lock of the tracking loop of target receiver onto it own code. It then drags the user position (or time) gradually to the planned magnitude, during the pull-off phase. After the pull-off phase, the spoofer has the complete control over the target receiver and can change the power / delay at its will. After planning of the spoofer profiles, the data from the field should be gathered.

#### Code phase alignment and power matching

After the recording, the data has to be aligned so that the epochs in the data should match. Coarse code phase alignment is done by aligning the navigation message data bit boundary. Data bit alignment produces accuracy in milliseconds. Fine code phase alignment is done by matching prompt-arm positions from the tracking loop of the receivers. Fine code alignment produces accuracy in terms of sample rate. After the code phase is aligned, signal power (carrier to noise ratio) is estimated and power on each receiver can be matched by scaling the respective data before mixing. Note that for synthetic signal generation for time-push scenario, code phase alignment, clock frequency and power matching is not required.

#### Clock frequency matching

Although the latest receiver and the recording modules have Temperature Controlled Crystal Oscillator (TCXO) as clock source, which has 0.5 ppm clock stability, but even this high accuracy produces spill over or short fall of approximately 8 samples in a 16.368 MHz sample clock in one second. In order to match the clocks, the code phase is to be matched again at the end of control phase using the same procedure as described in previous section. The code phase difference found at the end of control phase is only due to the clock differences of both recorder receivers. Based on the difference in the clock frequencies, the samples from the records having higher sampling rate are to be adjusted before mixing the files.

Another method of finding the clock frequency matching is by taking the difference of the receiver clock correction term calculated during the navigation solution calculation. The receiver calculates the receiver clock correction term at each measurement. The rate of change of clock correction corresponds to the frequency mismatch between the receiver and the satellite master clock. Similarly the difference in the rate of clock correction between the receivers corresponds to the frequency mismatch.

Note here that for a small alignment & control phase, the accuracy of clock matching term will be low, on the other hand, finding the clock matching through receiver clock correction requires calculating the navigation solution for complete period; which is not a difficult thing to do.

#### Power control (spoofer power profile)

In order to achieve the intermediate spoofing, the spoofer power should follow a pre-planned profile [7]. Fig. 1 shows one such profile that mimics intermediate attack. In this profile, the spoofer does not transmit any power during the alignment phase when target receiver parameters (given in Table 1) are being estimated. After the estimation, the spoofer starts controlling the target receiver by increasing its transmit power gradually so that it should not alarm the target receiver. The transmit power of the spoofer then stays to the 100% of the required power for the time the target is being spoofed.

**Table 1:** Spoofing plan with time period and distance between receivers in each spoofing phase.

Spoofing Phase	Time Period	Distance b/w Receivers
Alignment	0–0 s	0 m
Control	0–20 s	0 m
Pull-off	20–160 s	0–800 m
Capture	160–180 s	800 m

#### Signal mixing

After alignments and adjustments, the signals are mixed. The mixing equation is as follows;

(1) c[n]=γ[n]a[αn+k]+b[βn+l]

where *a*[n] and *b*[n] are the recorded signals from the field receivers. γ[n] is the power matching term based on the signal power and spoofer power profile, and (*α, β*) are clock correction scaling factor where one of them has to be 1 and other one is greater than 1. And (*k, l*) are code phase adjustment terms where one of them has to be zero and other to be greater than zero.

**Fig. 2:**
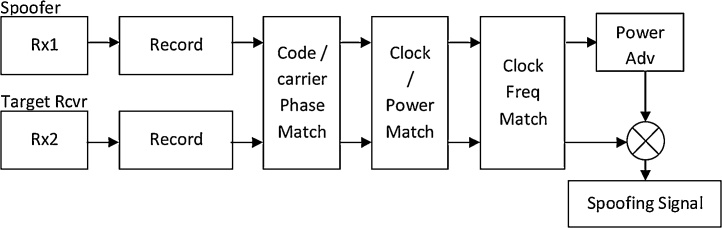
Flow chart showing process done to synthesize spoofing data.

The flow chart given in the Fig. 2 summarizes complete steps required to synthesize signal for a spoofing attack.

#### Spoofing detection

In order to verify the method, the results have to be checked through spoofing detection techniques. Several methods are available for detection of spoofing attack. We have adopted the analysis of signal ACF plot as it is a good indicator of the system under spoofing. In this method, the ACF is plotted and the spoofing is detected by the user through analysing the plots visually, looking for more than one peak in the plots. Furthermore, Shape Distortion based Spoofing metric (M_SD_) is also used to validate the results. The formula for the SD metric is given as under

(2) $$M_{SD}=\;\sqrt{\sum_{k=-K}^{K}{{{(ACF}_{meas}\left(\tau_{k}\right)}_{j}-\;{{ACF}_{Typ}\left(\tau_{k}\right)}_{j})}^{2}}$$

where

(3) $${R_{typ}\left(\tau_{i}\right)}_{j}=\;\left\{\begin{array}{cc} \left[1-\frac{\left|\tau_{i}\right|}{T_{c}}\right] & for\;\left[1-\frac{\left|\tau_{i}\right|}{T_{c}}\right]>\;\mu_{n_{j}}\\ \mu_{n_{j}} & other\;wise \end{array}\right\},$$

(4) $${R_{meas}\left(\tau_{i}\right)}_{j}=\;\sum_{n=\;0}^{N-1}r_{j}\left(n\right)c_{j}\left(n\right)e^{-j\left(2\pi f_{{IF}_{j}}n\right)},$$

and the threshold is calculated by

(5) $$\eta_{j}=C*\;{{\hat{N}}_{o}}_{j}$$

In Eqs. (2), (3), (4), (5), r(n) and c(n) are the digitized received signal and local generated replica PRN code respectively and $e^{-j\left(2\pi f_{{IF}_{j}}n\right)}$ is the local generated replica carrier for j^th^ satellite. N is number of samples in integration time T_s_ and defined as N = f_s_ * T_s_, where f_s_ is the sampling rate. μ_n_ is the calculated nominal values of ACF under the influence of noise. T_c_ is one chip time and k is the number of correlators on each side of the prompt correlator. ${{\hat{N}}_{o}}_{j}$ is the estimated noise power, C is constant calculated from un-spoofed data and $\eta_{j}$ is the threshold on signal distortion metric for j^th^ satellite for spoofing detection based on satellite’s noise power.

## Results

In order to verify the procedure, a position-push signal is synthesized through this procedure. Two similar raw signal recorders are used having MAX2769 Front-end, with sampling rate of 16.368Msps, and IF of 4.092Mhz. The signal recorders were equipped with TCXO with accuracy of about ±5 × 10^−7^ s/s. Data is collected for 180 s, with one receiver static and other moved for 800 m according to the plan given in Table 1.

### Data logging

**Fig. 3:**
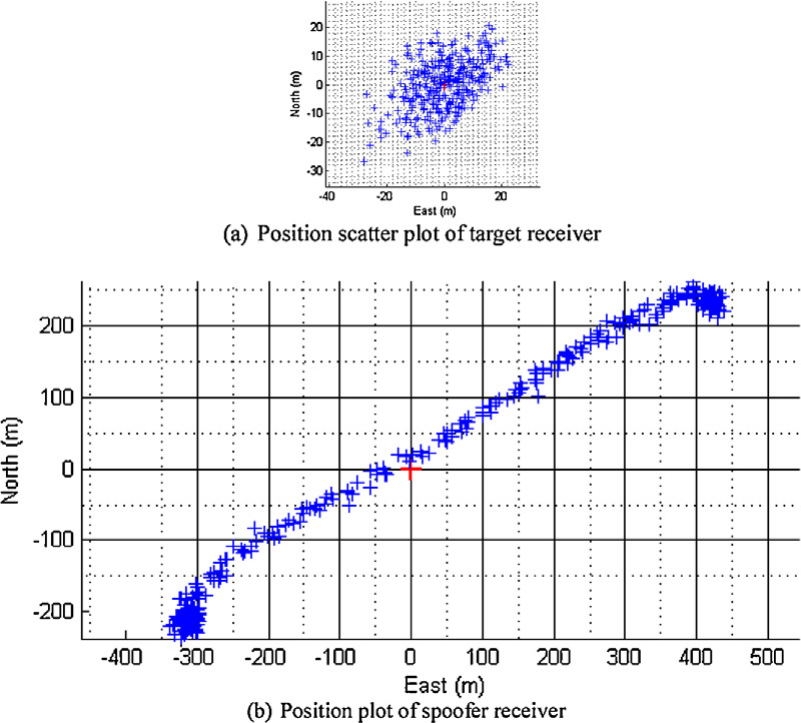
The navigation results of the recorded signals to launch a position push scenario for (a) stationary target receiver and (b) the spoofer intended position tracks.

After the signal data is logged using the receiver, the validity of the data is checked by processing the individual receiver records by a post processing Software Defined Receiver (SDR). The navigation results (position scatter plot) based on SDR tracking data of both receivers are depicted in Fig. 3.

Fig. 3 shows the position plots of the target and spoofer receivers to verify that the signals are recorded correctly and they are according to the plan given in Table 1, i.e. the target reciver whose position plot is given in Fig. 3(a) is stationary with a nominal error of 20 m due to normal envirnmental effects and the spoofer receiver moved to 800 m as we have planned position push scenario in which the spoofer will drag the position to this extent. The start point of the spoofer receiver is same as target receiver.

### Code phase alignment

**Fig. 4:**
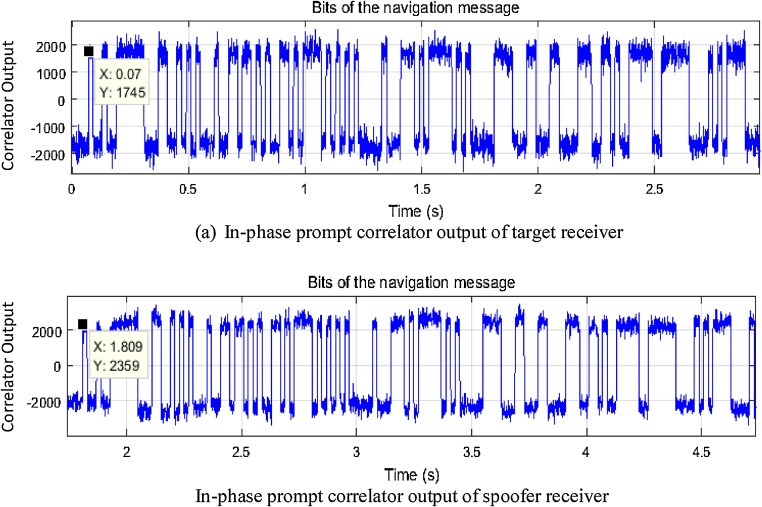
Navigation message plots for initial alignment of both receivers.

Due to different receivers involved, the start time for the recording of the receivers is not the same. Code phase alignment is to be done to match the start time. First the code phase is aligned coarsely for which the in-phase prompt correlator output is plotted, as given in Fig. 4. The in-phase prompt correlator output also corresponds to navigation data bits, which can be matched manually to align the signal records.

While tracking, each correlator produces output every millisecond (if the dwell time is one millisecond). The in-phase prompt correlator output contains the navigation bit information at 50 bits per second, which is evident from Fig. 4. As both receivers get the navigation information from the same satellite and the receivers are at the same location at start, they are to be in-sync with each other and any difference is due to the different recording start time.

The two signal records (target and spoofer) are visually aligned so that the same navigation bit pattern lines up. Time corresponds to same instance is noted which 70 ms and 1809 ms as seen from Fig. 4 (a) & (b). To get the precise phase information, the tracking results of processed data at the corresponding time is analysed. The tracking results contain the sample number corresponding to prompt tracking point for each correlator output. Using this information, the difference in code phase is found to be 28,461,822 samples that correspond to 1,738,869.86 μs time difference between start of the data recording on both receivers. Based on these estimations, *k* is found to 28,461,822 and *l* is taken as 0 for Eq. (1).

### Power / clock matching and control

To match the power, average of prompt envelop is calculated from in-phase and quadrature-phase components. The average of each channel is found equal, so the power matching term i.e. γ[n] is taken as required spoofer power advantage, for pull-off and control phases i.e. n>20. For creating the power profile, the spoofer power is varied according to Spoofer power profile given in Fig. 1 and Table 1.

For clock matching, the code phase alignment procedure is used again at time of 20 s, and it comes out that there is 85 sample differences. Through this observation, it is found that both crystals have a mismatch of 0.25,965 ppm. From these values α is found to be 1.00000025965 and *β* is taken as 1 for Eq. (1). To correct this problem the data is re-sampled accordingly.

Using the receiver clock error rate, the rate of change of clock is calculated for both of the receivers and found to be 0.920649 ppm and 0.666151 ppm; making the clock mismatch equals to 0.254498 ppm, which is a very close match to the code phase alignment method. It is recommended that if the navigation processing is done to the data; the receiver clock error matching should be used as it gives a direct and accurate value for the clock matching term.

### Spoofing output and verification

**Fig. 5:**
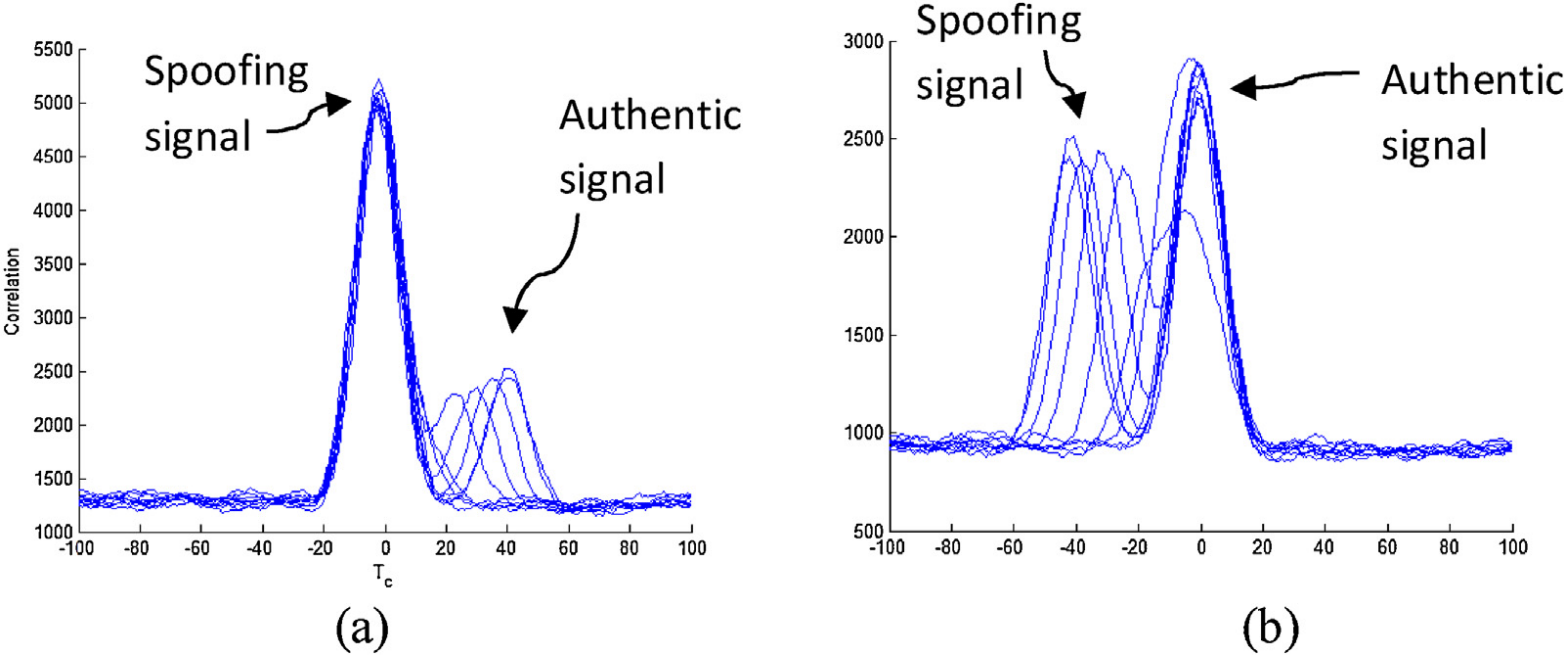
Autocorrelation Function plots for every 20 s of synthetically generated spoofing signal for (a) 3 dB spoofer power advantages and (b) -0.4 dB spoofer power advantage.

Using the parameters gathered in previous sections, the synthetically generated spoofing signal is produced by mixing the two signals as per Eq. (1). To verify the correct mixing through analysis of ACF, overlaid plots at different time are presented in Fig. 5 using a 100 ar m correlator.The ACF overlaid plots of one channel having 3 dB spoofer power advantage at 20 s interval are plotted in Fig. 5(a). The figure shows that in some of ACF plots there is only one peak visible and these are the ones that corresponds to the initial phases of the spoofing, when the receivers are physically close to each other. During the pull-off phase when two receivers are distant from each other, there are two peaks visible, and there is a clear deviation between the spoofing peak and the authentic peak. It is also shown that the receiver is locked on the spoofing signal due to its high power, and the authentic signal can be seen at the late side of the prompt tracking arm. Fig. 5(b) shows similar results but as the spoofer power advantage was low, the receiver continued to lock on the authentic signal and did not get spoofed. Spoofing is visible in these ACF plots on early side of the tracking arm. The spoofer power advantage is visible through the difference in the peaks produced by the authentic and spoofing signal.

**Fig. 6:**
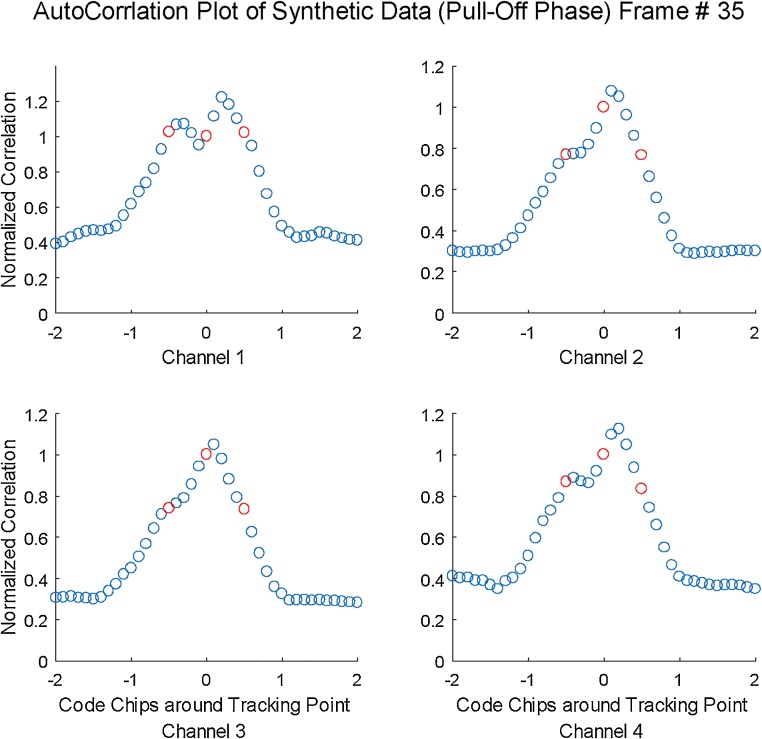
ACF plots of synthetically generated spoofing signal for 4 different channels at one time instance. A complete video depicting the Autocorrelation Function at each time instance is available with article.

The results of measured ACF are also presented in accompanied video that presents 4 channels of receivers being spoofed by the synthetic signal produced. Fig. 6 shows snapshot of the accompanied video, that shows the changed ACF at 35 s. Because of the position push scenario some of the channels are more delayed and some are less delayed.

**Fig. 7:**
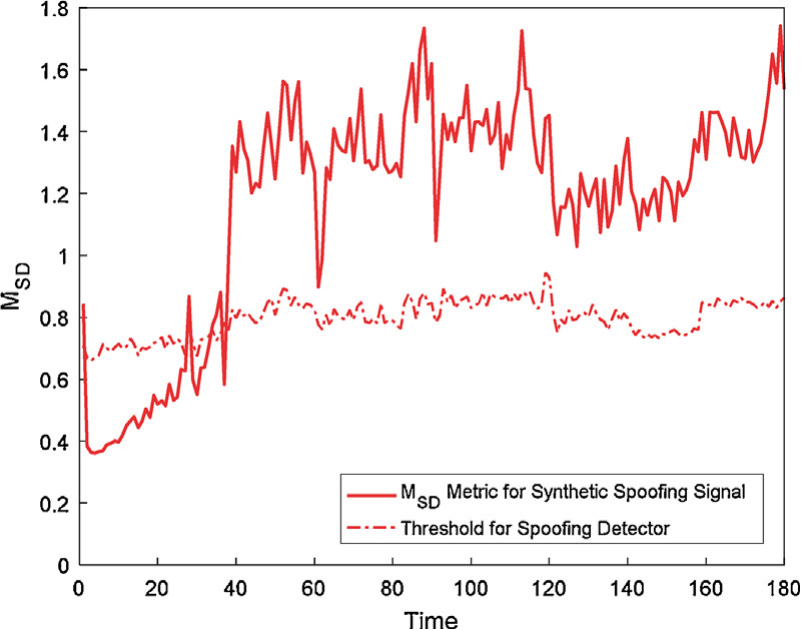
Plot of CSC Metric of the synthetically generated spoofing signal.

The data is further checked using SD metric based spoofing detector as given in Eq. (2). The M_SD_ based spoofing detector is very effective during the pull-off phase of the spoofing. Spoofing signal is generated for 3 dB spoofer power advantage and the M_SD_ metric is calculated and plotted in Fig. 7 along with the noise power based threshold using Eq. (5). The threshold is used by the detector to classify the signal based on the M_SD_ that it contains the spoofing signal or not. It can be seen that in the initial phase the signal is not classified as spoofing, but during the pull-off, the detector has classified the signal as spoofing. Successful detection of spoofing verifies that the spoofing attack is correctly synthesized in the signal.

## Conclusion

This paper has proposed a method of synthetically generating the signal with spoofing present in it. The method uses the data gathered through easily available recorder receivers to synthesize spoofing attack. The method has been verified by gathering and synthesizing real life data to get spoofing signal. The spoofing is visible in the ACF plots of the synthesized signal, and also confirmed through SD metric. Spoofing synthesized through this method is more realistic than simulation and can be done using very cheap and easily available equipment. The synthesized spoofing signals can be used to verify the spoofing detection and mitigation algorithms.
