# Supplementary material for: Synthetic GNSS spoofing data generation using field recorded signals
Source: MethodsX. 2018 Oct 9;5:1272–80. doi: 10.1016/j.mex.2018.10.004 (PMC6197700; doi:10.1016/j.mex.2018.10.004)
Supplement: Supplementary file 1 [file mmc1.docx]

**Additional information:**

**Introduction**

Global Navigation Satellite Systems (GNSS) are becoming primary source of Position, Navigation and Timing (PNT) for a variety of applications and has a big user base (Psiaki and Humphreys, 2016) (Ioannides et al., 2016). The GNSS signals are vulnerable to environmental effects, interference, jamming, and spoofing due to their low power and open signal structure (Khan and Ahmad, 2016) (Huang et al., 2016). With a greater user base and open architecture, it is becoming a tempting target for the attackers to spoof the GNSS signal. The spoofer can harm the system user by giving it Hazardously Misleading Information. However, the sophisticated and intermediate spoofing attack needs careful planning and positional knowledge about the target receiver. Furthermore, the spoofer has to follow a set of sequence of operations in order to launch the intermediate attack (Humphrey et al., 2012). Most of the text found in the literature for spoofing mitigation is based on the spoofing attack generated in simulation which does not account for natural environmental effects.

This paper focuses on generation of spoofing data synthetically by using signals recorded in the field. Two receivers are used to record the signals in which one receiver mimics target receiver and the other as attacker. The receiver that mimics target could be stationary or moving. The receiver that mimics attacker has to follow the course on which the spoofer wants the target receiver to be misled, according to the spoofer delay/position profile. After recording the data by the receivers, it is to be aligned for the time, corrected for the power and frequency mismatch due to different receiver clocks, and mixed according to the spoofer power profile. The generated spoofed data is similar to the standard spoofing attack data present in TEXBAT spoofing data shared by university of Texas at Austin (Humphreys et al., 2012).

The methods, sequence and specifications of spoofing is discussed later in details in the succeeding sections. Out of the three type of spoofing attacks, namely simplistic, intermediate and sophisticated, we have focused on the intermediate spoofing that is most deceitful practical method of spoofing. The spoofing sequence is necessary to break the lock of the receiver on authentic signal and make it lock on the counterfeit signal generated by the spoofer. The method presented in this paper takes care of the spoofing sequence aspect to mimic exact spoofing attack scenario. The paper also discusses different spoofing specifications that can be changed by a spoofer to launch a best possible attack on the target receiver. Synthesizing a spoofing attack gives flexibility to create variety of spoofing scenarios from the same data that have real environmental effects in it. Through this approach anti-spoofing methods can be tested by changing selective spoofing parameters on the same dataset.

**Spoofing Attack**

Spoofing is method of deceiving the target receiver through counterfeit signals that are similar to the authentic signal in every respect, i.e. carrier, code and navigation message values and their specifications (carrier / code phase, doppler, amplitude and navigation message bit boundary time). An intermediate or sophisticated spoofer needs the carefully planned spoofing parameters to launch of attack, as given in Table II.

**Table II:** Spoofing parameters

| Spoofing parameters |
| --- |
| Start time of the k^th^ C/A code period |
| Code and carrier phase at start time |
| Doppler frequency at start time |
| Signal amplitudes at start time |
| The current 3-dimensional position P and velocity V at start time. |

These parameters are varied according to the spoofer specifications; that are given in the table III (Humphreys et al., 2012).

**Table III:** Spoofer Specifications

| Specification | **Description** |
| --- | --- |
| Type | Simplistic/Intermediate/Sophisticated |
| Sequence | Control and pull-off phase time duration |
| Variation | Position or time-push |
| Magnitude | Total Variation in position or time during pull-off |
| Strength | Spoofer power advantage |

*How to Spoof a Receiver*

The Spoofer intending intermediate attack on a target receiver first estimates the critical parameters given in Table II, required for matching the counterfeit and genuine signal so that they appear at one point in the target receiver correlation function. In order to achieve this, it uses a receiver at its own location and should know the precise distance to the target receiver. Separate parameters for each satellite are estimated and given to the spoofer module that generates the data stream that includes the code, carrier and navigation data required for spoofing the target receiver. All data streams for each satellite are combined and adjusted for power and given to the transmitter. A control module looks after the coordination of the whole process.

Intermediate spoofers try to accurately estimate the code phase, frequency and navigation data bits of the target receiver (Humphreys et al., 2012) and secretly align the correlation peak to the genuine one. After the alignment phase, the counterfeit signal power is gradually increased until it begins to control the tracking loops of the target receiver. The counterfeit signal is then dragged away from the authentic signal. This process is called pull-off and it generates Auto-Correlation Function (ACF) distortion. Once the pull-off phase is complete and the receiver is locked on the spoofed signal away from the genuine signal this phase is known as the capture phase and the spoofer can now change the signal anyway it likes to send any fake information to the receiver (Humphreys et al., 2008). Figure 8 provides an overview of the spoofing sequence (Humphreys et al., 2012).

**Figure 8:** Phases of intermediate spoofing sequence.

*Time Push and Position Push*

While spoofing the satellite signal, if code phase on all of the channels are delayed for the same amount of time, the delay is catered in the navigation processing as the receiver clock error. The receiver clock gets an offset due to the clock error but the position solution is not affected. This method is termed as time-push. And when each satellite's code phase is delayed appropriately so that the navigation processing in the target receiver converges to the spoofer’s desired position (Humphreys et al., 2012), it is called the position push.

In Time-push, after the spoofed code phase is delayed from authentic signal on all of the channels at the same time, the spoofer is free to change the navigation message or change each channel separately. Time-push is more preferred method of launching attack. In Position Push, spoofed code phase is changed from authentic signal according to the phase requirement for the new position planned for the target receiver.

Spoofing signal

Authentic signal

Authentic signal

Spoofing signal


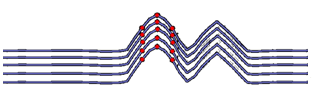

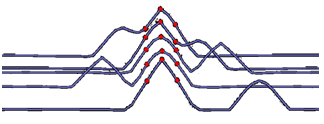


Auto Correlation Function

(a)

(a) (b)

**Figure 9:** Possible ACFs of different channels for (a) Time-Push and (b) Position-Push Scenario.

Figure 9 shows the possible ACFs of a spoofed signal showing the peak due to authentic signal and spoofed signal for different channels being tracked and spoofed in position push and time push scenario. Figure 9(a) shows that spoofer delays each channel for equal amount and tries to hide the attack as multipath by placing the non-tracking peak on the late side. Figure 9(b) shows that the spoofer delays each channel for different amount of time and on different side, so that the net effect produces the solution within the receiver at the spoofer desired point.

*Power Advantage of Spoofer*

The Spoofer power advantage is the ratio of spoofer power to the power of authentic signal from satellite, as received at the target receiver’s antenna.

*Hiding Spoofing Attack in Multipath*

The spoofer always tries to transmit the signal such that it is delayed from the authentic signal in the initial phases (alignment and control) and is earlier in the later phases (pull-off and capture). The reason for delaying in the initial spoofing phases is because the receiver is locked on the authentic signal and a delayed spoofing signal appears to be similar to multipath signal to it. For the later phase, the receiver is locked on the spoofing signal and dragging it earlier makes authentic signal appear to receiver like multipath signal.
